# Metafounders May Reduce Bias in Composite Cattle Genomic Predictions

**DOI:** 10.3389/fgene.2021.678587

**Published:** 2021-08-20

**Authors:** Sabrina Kluska, Yutaka Masuda, José Bento Sterman Ferraz, Shogo Tsuruta, Joanir Pereira Eler, Fernando Baldi, Daniela Lourenco

**Affiliations:** ^1^Departamento de Zootecnia, Universidade Estadual Paulista Júlio de Mesquita Filho, Jaboticabal, Brazil; ^2^Department of Animal and Dairy Science, University of Georgia, Athens, GA, United States; ^3^Departamento de Medicina Veterinaìria, Universidade de São Paulo, Pirassununga, Brazil

**Keywords:** genomic selection, inflation, Montana cattle, single-step genomic BLUP, unknown parent groups

## Abstract

Metafounders are pseudo-individuals that act as proxies for animals in base populations. When metafounders are used, individuals from different breeds can be related through pedigree, improving the compatibility between genomic and pedigree relationships. The aim of this study was to investigate the use of metafounders and unknown parent groups (UPGs) for the genomic evaluation of a composite beef cattle population. Phenotypes were available for scrotal circumference at 14 months of age (SC14), post weaning gain (PWG), weaning weight (WW), and birth weight (BW). The pedigree included 680,551 animals, of which 1,899 were genotyped for or imputed to around 30,000 single-nucleotide polymorphisms (SNPs). Evaluations were performed based on pedigree (BLUP), pedigree with UPGs (BLUP_UPG), pedigree with metafounders (BLUP_MF), single-step genomic BLUP (ssGBLUP), ssGBLUP with UPGs for genomic and pedigree relationship matrices (ssGBLUP_UPG) or only for the pedigree relationship matrix (ssGBLUP_UPGA), and ssGBLUP with metafounders (ssGBLUP_MF). Each evaluation considered either four or 10 groups that were assigned based on breed of founders and intermediate crosses. To evaluate model performance, we used a validation method based on linear regression statistics to obtain accuracy, stability, dispersion, and bias of (genomic) estimated breeding value [(G)EBV]. Overall, relationships within and among metafounders were stronger in the scenario with 10 metafounders. Accuracy was greater for models with genomic information than for BLUP. Also, the stability of (G)EBVs was greater when genomic information was taken into account. Overall, pedigree-based methods showed lower inflation/deflation (regression coefficients close to 1.0) for SC14, WWM, and BWD traits. The level of inflation/deflation for genomic models was small and trait-dependent. Compared with regular ssGBLUP, ssGBLUP_MF4 displayed regression coefficient closer to one SC14, PWG, WWM, and BWD. Genomic models with metafounders seemed to be slightly more stable than models with UPGs based on higher similarity of results with different numbers of groups. Further, metafounders can help to reduce bias in genomic evaluations of composite beef cattle populations without reducing the stability of GEBVs.

## Introduction

Single-step genomic BLUP (ssGBLUP) has been widely used for genomic evaluation in domestic species such as dairy and beef cattle, swine, and chicken (Chen et al., 2011; Tsuruta et al., 2013; Lourenco et al., 2015b; Song et al., 2017). The main advantage of this method is the combination of genotyped and non-genotyped animals in the same analysis, possibly providing less biased and more accurate predictions than multistep methods (Chen et al., 2011; Legarra et al., 2014). However, the realized relationship matrix (**H**) used in ssGBLUP was developed under assumptions that may not hold in practice and may result in biased genomic estimated breeding value (GEBV), especially when pedigree information is missing for genotyped animals (Misztal et al., 2013). In such a case, incompatibilities between the genomic (**G**) and pedigree (**A**) relationship matrices are observed (Misztal et al., 2013). Incompatibilities are also related to different base population assumptions for each source of information. While the base population for **A** is assumed to be the founders of the pedigree, the base for **G** is frequently the current genotyped population because **G** is most often centered using current allele frequencies (Vitezica et al., 2011). Several approaches have been proposed to solve the incompatibility between **G** and **A** in ssGBLUP, namely, truncation of pedigree to the most recent generations (Lourenco et al., 2014), scaling parameters for **G** and **A** (Aguilar et al., 2010), and different ways to construct **G** (Chen et al., 2011; Simeone et al., 2012).

The incompatibility between **G** and **A** may be intensified in crossbred or multibreed populations because the allele frequencies used to center and scale **G** are usually based on means across genotyped animals in the population (Lourenco et al., 2016). An additional problem arises in composite breeds that are formed of two or more breeds, and sometimes their crossbreds, causing the base population to be heterogeneous. Correctly modeling differences in base populations can result in less biased genomic predictions and more appropriate selection decisions for such populations (Macedo et al., 2020).

In practice, pedigrees used in genetic evaluations may trace back to several base populations that are assumed to be unrelated because this information is unavailable. However, base animals may be related in **G** because of its identity-by-state nature. If this is the case, **G** and **A** will have unbalanced information, causing GEBV to be biased (Legarra et al., 2015). In addition to missing information at the “beginning” of the pedigree, animals from different generations may have missing pedigree information (Tsuruta et al., 2019). If not correctly modeled, founders, and animals with missing pedigree will have their breeding values regressed toward zero, which is not realistic because populations are under selection (Legarra et al., 2015). Quaas (1988) and Westell et al. (1988) proposed the use of unknown parent groups (UPGs) to overcome problems related to missing pedigree information. The UPGs permit modeling differences in genetic merit across classes of missing parents such as year of birth, sex, and country of origin. Additionally, UPGs may be used to account for differences among breeds (Legarra et al., 2007). However, the UPG approach still assumes that the base populations are unrelated, which is often not true.

To solve this issue, Legarra et al. (2015) recently proposed the concept of metafounders (MFs), which are pseudo-individuals that act as proxies for animals in base populations. When MFs are used, base individuals can be related though the pedigree, improving the compatibility between **G** and **A**. Metafounders can be interpreted as a generalization of UPGs, but with relationships among animals within and across base populations (Legarra et al., 2015). According to Garcia-Baccino et al. (2017), the inclusion of MFs in the model reduces the bias of genomic predictions without loss of accuracy. The assumption that pedigree founders are fully unrelated is voided in the MF approach. There are a few studies evaluating the performance of ssGBLUP with MFs in real crossbred populations (Xiang et al., 2017), but none in composite populations.

Montana is a beef cattle composite breed where the base population is composed primarily of four different biological types, defined as the NABC system. Biological type N refers to animals from *Bos taurus indicus* breeds, A to animals from *Bos taurus taurus* breeds adapted to the tropics, B to British *Bos taurus taurus* animals, and C to taurine animals from continental Europe (Ferraz et al., 2002). The current genomic evaluation system for Montana beef cattle does not account for a heterogeneous base population, but it fits breed proportions as covariates in the model, which should be at least in theory equivalent to models with UPGs in **A** matrices. Thus, the objective of this study was to evaluate the use of MFs and UPGs to model the base population used for genomic evaluations in a Montana composite beef cattle population.

## Materials and Methods

Animal Care and Use Committee approval was not obtained for this study because the dataset was obtained from an existing database.

### Phenotypic and Genomic Data

Data for this study were from the Montana Composto Tropical^®^—*CFM Leachman Pecuária Ltda*. breeding program. The dataset included phenotypes for scrotal circumference and growth traits. Pedigree information was available for 680,551 purebred, intermediate crossbred, and composite animals. A total of 4,212 sires and 192,619 dams were the founders of this breed according to the Montana breed association. Traits included scrotal circumference at 14 months of age (SC14, cm), post weaning weight gain (PWG, kg; calculated as the difference between yearling weight adjusted to 420 days of age and weaning weight adjusted to 205 days of age), weaning weight (WW, kg; weaning weight adjusted to 205 days of age), and birth weight (BW, kg). Phenotypic records deviating from the mean of contemporary groups ±3 standard deviations and contemporary groups with less than five records were removed. Contemporary groups for BW and WW were based on farm, year and season of birth, sex, and management group classes. Contemporary groups for SC14 and PWG were defined using farm, year of birth, and weaning management group classes. After data editing, a total of 49,541 phenotypic records for SC14, 96,994 for PWG, 325,014 for WW, and 264,981 for BW were available. The heritability estimates were 0.25 for SC14, 0.09 for PWG, 0.23 for WW, and 0.22 for BW. Heritabilities for all models were estimated using a BLUP model without UPGs or MFs because UPGs had a minor influence on estimated genetic parameters (Theron et al., 2002). The MF approach required the use of scaled variance components. This was accomplished within the BLUPF90 program.

A total of 1,899 animals were genotyped for 30k, 35k and 770k, respectively single-nucleotide polymorphism (SNPs). Subsequently, all genotypes were imputed to the *Neogen Gene Seek^®^ Genomic Profiler* (GGP) commercial panel with approximately 30k SNP markers using the FImpute software (Sargolzaei et al., 2014). After SNPs with minor allele frequencies below 5%, call rates lower than 90%, and departures from the Hardy–Weinberg equilibrium (difference between expected and observed frequencies of heterozygous) greater than 0.15 and with unknown position or located on sex chromosomes were removed, the edited genotype file contained 27,373 SNPs from 1,797 animals born between 1999 and 2016.

### Statistical Analysis

Both UPGs and MFs were used to model the heterogeneous Montana base population. A four-trait model was applied to the pedigree BLUP (BLUP); pedigree BLUP with UPGs (BLUP_UPG); pedigree BLUP with MFs (BLUP_MF); ssGBLUP; single-step GBLUP with UPGs for **A**,**G**, and the pedigree relationship matrix for genotyped animals (**A**_22_) (ssGBLUP_UPG) or only for **A** and **A**_22_(ssGBLUP_UPGA); and single-step GBLUP with MFs (ssGBLUP_MF). The model without UPGs was as follows:

$$\text{y}=\text{Xb}+{\text{Z}}_{1}\text{u}+{\text{Z}}_{2}\text{m}+{\text{Z}}_{3}\text{c}+\text{e},$$

where **y** is the vector of phenotypes for each trait (SC14, PWG, WW, and BW); **b** is the vector of contemporary group and age of dam classes, and biological type composition covariates for models without UPGs or MFs, non-additive effects for total maternal heterozygosity (H_m_), non-additive effects for direct heterozygosity (N × A, N × B, N × C, A × B, A × C, and B × C), and age at recording for SC14; **u** is the vector of direct additive genetic effects for SC14, PWG, WW (WWD), and BW (BWD); **m** is the vector of maternal additive genetic effects for WW (WWM) and BW (BWM); **c** is the vector of maternal permanent environmental effects for WW and BW; **e** is the vector of residuals; and **X**, **Z**_1_,**Z**_2_, and **Z**_3_ are incidence matrices relating phenotypic records in vector **y** to effects in vectors **b**, ***u***, **m**, and **c**, respectively.

When UPGs were added to pedigree-based BLUP and ssGBLUP evaluations, a **Z**_1_**Qg** term was added to the model, as follows:

$$\text{y}=\text{Xb}+{\text{Z}}_{1}\text{u}+{\text{Z}}_{2}\text{m}+{\text{Z}}_{3}\text{c}+{\text{Z}}_{1}\text{Qg}+\text{e},$$

where **Q** is an incidence matrix relating animals in vector ***u*** to UPGs in vector **g**. Traditional EBV and GEBV for UPG models were calculated as follows:

(G)EBV=Qg+u.

The UPGs for ssGBLUP were modeled in two different ways. Firstly, UPGs were applied to all pedigree-based and genomic relationship matrices that compose **H** (Misztal et al., 2013). The resulting model was defined as ssGBLUP_UPG. Matrix **H**^−1^ for ssGBLUP_UPG (${\text{H}}_{UPG}^{*}$) was constructed as follows:

$${\text{H}}_{UPG}^{*}={\text{A}}^{*}+\left[\begin{array}{ccc} 0\; & 0\; & 0\\ 0\; & {\text{G}}^{-1}-{\text{A}}_{22}^{-1}\; & -\left({\text{G}}^{-1}-{\text{A}}_{22}^{-1}\right)\text{Q}\\ 0\; & -{\text{Q}}^{\prime }\left({\text{G}}^{-1}-{\text{A}}_{22}^{-1}\right)\; & {\text{Q}}^{\prime }\left({\text{G}}^{-1}-{\text{A}}_{22}^{-1}\right)\text{Q} \end{array}\right],$$

where A^*^ is the inverse of the pedigree relationship matrix with UPGs constructed with the QP transformation (Quaas, 1988), and **G** is the genomic relationship matrix computed as in Van Raden (2008) with allele frequencies from the current genotyped population. Relationships in **G** are identical by state; thus, **G** is unaffected by missing pedigree (Tsuruta et al., 2019). Because of this, a second formulation used UPGs only in **A** and **A**_22_; this model was called ssGBLUP_UPGA. The **H**^−1^for ssGBLUP_UPGA (${\text{H}}_{UPGA}^{*}$) was constructed as follows:

$${\text{H}}_{UPGA}^{*}={\text{A}}^{*}+\left[\begin{array}{ccc} 0\; & 0\; & 0\\ 0\; & {\text{G}}^{-1}-{\text{A}}_{22}^{-1}\; & -{\text{A}}_{22}^{-1}\text{Q}\\ 0\; & -{\text{Q}}^{\prime }{\text{A}}_{22}^{-1}\; & {\text{Q}}^{\prime }{\text{A}}_{22}^{-1}\text{Q} \end{array}\right].$$

A third approach used to model the heterogeneous Montana base population was MFs (Legarra et al., 2015). The model with MFs was defined as ssGBLUP_MF. Matrix **A** in ssGBLUP_MF was modified to be compatible with **G** centered with allele frequencies of 0.5 (**G**_05_). The **H**^−1^ for ssGBLUP_MF (**H**^Γ−1^) was computed as follows:

$${\text{H}}^{\Gamma -1}={\text{A}}^{\Gamma -1}+\left[\begin{array}{ccc} 0\; & 0\; & 0\\ 0\; & {\text{G}}_{05}^{-1}-{\text{A}}_{22}^{\Gamma -1}\; & 0\\ 0\; & 0\; & 0 \end{array}\right],$$

where **A**^Γ−1^ and ${\text{A}}_{22}^{\Gamma -1}$ are **A**^−1^ and ${\text{A}}_{22}^{-1}$ modified with **Γ**, the matrix of relationships among MFs that accounts for similarities across base populations (Legarra et al., 2015). The **Γ** matrix included pedigree and SNP markers and was computed using a generalized least squares (GLS) approach (Garcia-Baccino et al., 2017) with the gammaf90 program of the BLUPF90 software suite (Misztal et al., 2014). All other computations in this study were also done with programs from the BLUPF90 software suite.

### Assignment of Metafounders to Montana Composite Cattle

The MFs and UPGs were assigned based on the biological type of the animals (N, A, B, and C). The Montana composite beef cattle is formed by biological type clusters according to likeness, physiology, production, and reproduction levels using a combination of both *B. taurus indicus* and *B. taurus taurus* animals. The base population is mainly composed of the four biological types (N, A, B, and C). Intermediate crosses between base animals can also be used in different proportions to generate composites. Table 1 presents numbers of missing parents, pedigree animals, and genotyped animals for each genetic group defined based on the biological types of Montana cattle.

**TABLE 1:** Numbers of missing parents, pedigree animals, and genotyped animals per genetic group.

Genetic group	GG4^1^	GG10^2^	Number of animals in the pedigree^3^	Number of genotyped animals
N	367,737	257,939	178,845	0
A	62,753	7,214	3,658	0
B	43,568	31,572	14,978	0
C	7,058	17,370	712	0
NA, AB, AC, BC	–	8,588	6,151; 1,797; 33; 3,197	1; 6; 0; 0
NB	–	25,583	98,377	0
NC	–	20,590	53,504	0
NAB, NAC, ABC	–	39,319	99,447; 31,369; 3,035	217;31; 18
NBC	–	22,316	29,231	0
NABC	–	59,625	155,876	1,626
Without breed information			340	0

*MF, metafounder; UPG, unknown parent group. ^1^Genetic group four is based on the main biological types (N, A, B, and C). ^2^Genetic group 10 is based on the main biological types (N, A, B, and C) and intermediate crossbreds (NA, AB, AC, BC, NB, NC, NAB, NAC, ABC, NBC, and NABC, where NA, AB, AC, and BC were combined into one group, as well as NAB, NAC, and ABC). ^3^Number of animals in the raw dataset before the addition of MF or UPG.*

Two genetic group definitions were used for both MFs and UPGs. Firstly, only four groups (GG4) that traced animals back to their N, A, B, and C founders were assigned. Missing parents of animals with a higher proportion of a particular biological type were assumed to be from that biological group. For example, a missing parent of an animal with a higher proportion of N was assigned to biological group N. Breed composition of almost all animals in the dataset was either known or estimated. Even when one parent was unknown, its breed proportion was inferred through the breed composition of its progeny. Further, when the two parents of an animal with known breed composition were unidentified, breed composition of the animal was assigned to both parents.

In the second group definition, 10 groups (GG10) that traced animals back to their main biological type and their two-way, three-way, and four-way crossbreds were assigned. Initially, groups were represented by N, A, B, C, NA, AB, AC, BC, NB, NC, NAB, ABC, NAC, NBC, and NABC. However, the number of genotyped animals in the Montana population is small; thus, groups with only a few animals were merged to avoid computational problems when estimating group effects. Groups NA, AB, AC, and BC were merged into a single group, as well as NAB, NAC, and ABC. The breed proportion of an animal and its known parents were taken into account when assigning groups. Thus, if an animal of biological type NA had a known parent of biological type A, its missing parent was assigned to group N. When both parents were unknown, only the breed proportion of animal was taken into account for group assignments. For example, if the biological type of an animal with unknown parents was NA, one parent was assumed to come from group N and the other parent from group A. Numbers of animals in GG4 and GG10 are shown in Table 1.

### Evaluation of Model Performance

The linear regression (LR) validation method (Legarra and Reverter, 2018) was used to evaluate model performance. The validation group was composed of 436 genotyped animals born from June to December of 2016 (year of the youngest genotyped animals with complete data), which had their phenotypes removed from the evaluation together with phenotypes from contemporaries. This will be referred to as the reduced dataset and will be represented by subscript *r*. The total number of phenotypic records per trait in the reduced dataset was 47,949 for SC14, 93,701 for PWG, 317,703 for WW, and 257,368 for BW. The complete dataset, identified with subscript *c*, included 49,541 phenotypes for SC14, 96,994 for PWG, 325,014 for WW, and 264,981 for BW. This dataset was used as a benchmark for validations. All evaluation models were run with both reduced and complete datasets, and all computations were done with programs from the BLUPF90 software suite (Misztal et al., 2014).

The estimators of the LR method were calculated based on Legarra and Reverter (2018) and Macedo et al. (2020). The accuracy of (G)EBV for validation animals was calculated as $\rho_{c,r}=\sqrt{\frac{cov\left({\hat{u}}_{c},{\hat{u}}_{r}\right)}{\left(1-\bar{F}\right)\sigma_{u}^{2}}}$, where *cov* is the sample covariance, $\hat{\text{u}}$ is the vector of (G)EBV, $\bar{F}$ is the average inbreeding coefficient for validation animals, and $\sigma_{u}^{2}$ is the additive genetic variance. The correlation between ${\hat{\text{u}}}_{c}\text{and}{\hat{\text{u}}}_{r}$, i.e., $cor_{c,r}=cor\left({\hat{\text{u}}}_{c},{\hat{\text{u}}}_{r}\right)$, which assesses the association between (G)EBVs obtained with the complete and reduced datasets, was used as a measure of consistency between subsequent evaluations. A high *cor*_*c*,*r*_ indicates that adding more data will result in few (G)EBV changes; thus, the reduced dataset is a good predictor of the complete dataset in this case. Inflation/deflation of (G)EBV was assessed as the deviation of the regression coefficient (b_1_) from 1, where b_1_ was obtained from the regression of ${\hat{\text{u}}}_{c}$ on ${\hat{\text{u}}}_{r}$:

$${\hat{\text{u}}}_{c}={\text{b}}_{0}+{\text{b}}_{1}{\hat{\text{u}}}_{r}.$$

Another estimator used to measure the model performance was bias, calculated as $\mu_{cr}=\bar{{\hat{u}}_{r}}-\bar{{\hat{u}}_{c}}$, where μ_*cr*_ has an expected value of zero if the evaluation is unbiased.

## Results

### Unknown Parent Group Solutions and Descriptive Statistics of Genomic Matrices With and Without Metafounders

Table 2 presents UPG solutions for all genomic models and traits. Overall, the UPG solutions for models with 10 groups were higher than for models with four groups. In addition, UPG solutions from models where UPGs were only taken into account in **A** matrices (ssGBLUP_UPGA) were higher than those from models with UPGs in all relationship matrices (ssGBLUP_UPG).

**TABLE 2:** Unknown parent group (UPG) solutions from complete (c) and reduced (r) data for genomic models with four or 10 groups added to all ssGBLUP relationship matrices (ssGBLUP_UPG) and to only pedigree-based relationship matrices (ssGBLUP_UPGA).

		UPG solutions							
UPG	Model	SC14_*c*_	SC14_*r*_	PWG_*c*_	PWG_*r*_	WW_*c*_	WW_*r*_	BW_*c*_	BW_*r*_
1	ssGBLUP_UPG4	0.07	–0.46	0.45	–0.66	–0.72	–1.59	–0.41	0.20
1	ssGBLUP_UPG10	–0.90	–0.39	–0.13	–0.02	–3.30	–2.30	0.37	–0.16
1	ssGBLUP_UPGA4	–1.03	–0.28	0.39	12.57	2.90	9.70	–0.12	1.27
1	ssGBLUP_UPGA10	–1.43	–0.24	2.80	0.39	–4.76	–0.88	0.63	–0.53
2	ssGBLUP_UPG4	0.51	–0.07	1.16	–0.29	–1.04	–1.25	–0.69	0.10
2	ssGBLUP_UPG10	–0.12	–0.36	–1.89	–1.81	3.09	2.80	–0.09	0.24
2	ssGBLUP_UPGA4	–0.97	–0.28	–0.80	16.69	2.11	9.27	–0.68	–1.97
2	ssGBLUP_UPGA10	–4.09	–0.53	14.87	23.30	–5.72	–12.35	0.72	1.78
3	ssGBLUP_UPG4	1.01	0.37	1.57	0.09	–0.03	–0.03	–1.09	–0.15
3	ssGBLUP_UPG10	1.03	0.47	–0.96	–1.35	–0.18	–0.44	–1.04	0.02
3	ssGBLUP_UPGA4	–0.06	0.43	0.98	13.49	2.58	10.73	–1.05	–2.10
3	ssGBLUP_UPGA10	–0.18	–0.01	8.46	3.78	–5.11	–3.16	–1.12	0.79
4	ssGBLUP_UPG4	0.80	0.14	0.00	–1.59	1.02	0.83	–1.17	0.14
4	ssGBLUP_UPG10	1.33	0.87	–3.58	–3.80	9.75	8.16	0.48	1.41
4	ssGBLUP_UPGA4	–0.45	0.06	–0.17	14.35	6.27	11.09	–1.53	–2.23
4	ssGBLUP_UPGA10	–0.06	0.40	6.51	2.18	5.90	5.01	0.43	1.79
5	ssGBLUP_UPG10	–0.32	–0.81	–0.83	–1.54	–1.99	–3.39	–0.48	–0.19
5	ssGBLUP_UPGA10	–0.93	–1.20	5.34	–0.12	–4.71	–2.64	–0.26	–0.13
6	ssGBLUP_UPG10	0.16	0.03	–3.44	–3.70	–0.28	–1.04	0.16	0.15
6	ssGBLUP_UPGA10	–0.58	–0.23	3.09	–1.81	–2.70	–1.41	0.34	0.02
7	ssGBLUP_UPG10	0.21	0.17	–2.19	–2.31	–2.56	–2.39	0.76	0.62
7	ssGBLUP_UPGA10	–0.58	–0.01	3.66	0.51	–7.29	–2.39	1.12	0.46
8	ssGBLUP_UPG10	–0.21	–0.59	1.29	1.29	–0.64	–1.65	0.18	0.37
8	ssGBLUP_UPGA10	–0.74	0.90	6.83	2.83	–3.88	–2.30	0.35	0.40
9	ssGBLUP_UPG10	0.02	–0.19	–2.18	–2.03	–2.77	–5.78	0.33	0.42
9	ssGBLUP_UPGA10	–0.57	–0.46	4.39	–0.97	–6.08	–6.85	0.59	0.24
10	ssGBLUP_UPG10	–0.15	–0.58	2.71	2.83	0.05	–1.22	0.17	0.34
10	ssGBLUP_UPGA10	–0.58	–0.86	8.09	3.93	–3.01	–1.68	0.34	0.33

Table 3 presents descriptive statistics for diagonal and off-diagonal elements from **G** and **A**_22_ with and without MFs. The inclusion of MFs in the pedigree-based relationship matrix and the assumption of allele frequencies equal to 0.5 causes an upward shift in the means of **A**_22_ and **G**. Noticeably, diagonal and off-diagonal element values for **A**_22_ and **G** were virtually identical when the number of MFs was increased from four to 10.

**TABLE 3:** Descriptive statistics for diagonal and off-diagonal elements of genomic matrices required for genomic evaluations with and without metafounders.

Matrix^1^	Parameter				
	Mean	Minimum	Maximum	Variance	Correlation between all elements of G and A
**Diagonal**					
**A**_22_	1.01	1.00	1.28	0.00	
**A**_22_(**Γ**_4_)	1.07	1.00	1.33	0.00	
**A**_22_(**Γ**_10_)	1.07	1.00	1.34	0.00	
**G**	1.01	0.47	1.23	0.00	0.10
**G**(**Γ**_4_)	1.18	0.46	1.43	0.00	0.65
**G**(**Γ**_10_)	1.18	0.46	1.43	0.00	0.67
**Off-diagonal**					
**A**_22_	0.03	0.00	0.75	0.00	
**A**_22_(**Γ**_4_)	0.15	0.00	0.83	0.00	
**A**_22_(**Γ**_10_)	0.15	0.00	0.83	0.00	
**G**	0.03	−0.14	0.94	0.00	0.79
**G**(**Γ**_4_)	0.35	0.00	1.13	0.02	0.79
**G**(**Γ**_10_)	0.35	0.00	1.13	0.02	0.81

*MF, metafounder. ^1^**A**_22_, numerator relationship matrix for genotyped animals; **G**, genomic relationship matrix; **Γ**_4_, matrix of relationships among MF using data from 4 MF; **Γ**_10_, matrix of relationships among MF using data from 10 MF.*

### Relationships Within and Across Metafounders (Γ)

The relationships within MFs (diagonals of the **Γ** matrix) were smaller than one, and those between MFs (off-diagonals of the **Γ** matrix) were different from zero in both scenarios (GG4 and GG10). Relationships within MFs in GG4 are presented in **Γ**_4_ below and ranged from 0.15 to 0.38, whereas the relationship across MFs ranged from 0.09 to 0.18.

$$\Gamma_{4}=\left[\begin{array}{cccc} 0.19\; & 0.11\; & 0.09\; & 0.09\\ & 0.15\; & 0.13\; & 0.13\\ & & 0.24\; & 0.18\\ & & & 0.38 \end{array}\right].$$

Overall, relationships within MFs in GG10 were greater than those in GG4 and ranged from 0.15 to 0.65 (see **Γ**_10_ below). Relationships across MF for GG10 ranged from −0.11 to 0.23 as opposed to all positive values for GG4. In particular, relationships between MFs1 and 3 (biological types N and B) and between 1 and 4 (biological types N and C) showed negative values in GG10. The relationship between MFs1 and 2 (biological types N and A) in GG10 was close to zero.

$$\Gamma_{10}=\left[\begin{array}{cccccccccc} 0.59\; & 0.02\; & -0.11\; & -0.08\; & 0.02\; & 0.09\; & 0.18\; & 0.08\; & 0.07\; & 0.10\\ & 0.21\; & 0.15\; & 0.12\; & 0.15\; & 0.10\; & 0.09\; & 0.14\; & 0.13\; & 0.13\\ & & 0.39\; & 0.23\; & 0.20\; & 0.20\; & 0.11\; & 0.15\; & 0.21\; & 0.13\\ & & & 0.65\; & 0.16\; & 0.15\; & 0.07\; & 0.13\; & 0.17\; & 0.13\\ & & & & 0.48\; & 0.16\; & 0.13\; & 0.14\; & 0.16\; & 0.13\\ & & & & & 0.48\; & 0.15\; & 0.15\; & 0.20\; & 0.12\\ & & & & & & 0.57\; & 0.12\; & 0.16\; & 0.12\\ & & & & & & & 0.19\; & 0.14\; & 0.13\\ & & & & & & & & 0.35\; & 0.13\\ & & & & & & & & & 0.15 \end{array}\right].$$

### Accuracy and Stability of (Genomic) Estimated Breeding Value

Table 4 shows accuracies of direct and maternal (G)EBV for the 436 validation animals. Accuracies of direct (G)EBV were largely similar across pedigree-based models and across genomic models for all traits. On the other hand, accuracies of maternal (G)EBV were higher for pedigree-based models with UPGs or MFs. Adding UPGs to **A** and **A**_22_ (ssGBLUP_UPGA) showed greater changes in accuracy for PWG when compared with other genomic models. The accuracy of (G)EBVs ranged from 0.38 to 0.50 for SC14, 0.10 to 0.79 for PWG, 0.35 to 0.51 for WWD, 0.30 to 0.64 for WWM, 0.47 to 0.58 for BWD, and 0.31 to 0.44 for BWM. Higher accuracies were observed when genomic information was added to the model, except for PWG that exhibited mostly minor changes. Inclusion of either four or 10 MFs in ssGBLUP models decreased accuracies (0.02 to 0.08); however, accuracies were still higher than corresponding values in BLUP models for all traits, except for PWG and ssGBLUP_MF10. Conversely, addition of UPGs to BLUP and ssGBLUP models yielded maternal (G)EBV accuracies that were either similar or higher than those from the original models, except for ssGBLUP_UPGA4 and WWM.

**TABLE 4:** Accuracies of direct and maternal (G)EBV and correlations between direct and maternal (G)EBV for validation animals from the complete and reduced datasets using various models with and without genetic groups.

		Accuracy^1^						Correlation^2^				
Model	SC14	PWG	WWD	WWM	BWD	BWM	SC14	PWG	WWD	WWM	BWD	BWM
BLUP	0.39	0.45	0.35	0.30	0.47	0.31	0.64	0.75	0.48	0.88	0.66	0.90
BLUP_UPG4	0.39	0.45	0.36	0.56	0.51	0.35	0.64	0.75	0.50	0.96	0.66	0.90
BLUP_UPG10	0.39	0.40	0.36	0.57	0.48	0.37	0.64	0.71	0.50	0.96	0.67	0.86
BLUP_MF4	0.38	0.44	0.35	0.51	0.50	0.44	0.64	0.75	0.48	0.95	0.68	0.93
BLUP_MF10	0.38	0.40	0.36	0.49	0.49	0.39	0.66	0.74	0.50	0.95	0.70	0.93
ssGBLUP	0.48	0.43	0.44	0.62	0.57	0.34	0.74	0.74	0.67	0.94	0.81	0.92
ssGBLUP_UPG4	0.48	0.43	0.44	0.62	0.57	0.38	0.75	0.75	0.67	0.98	0.81	0.92
ssGBLUP_UPG10	0.50	0.43	0.44	0.62	0.58	0.37	0.75	0.74	0.67	0.97	0.81	0.86
ssGBLUP_UPGA4	0.50	0.10	0.51	0.61	0.56	0.40	0.75	0.02	0.72	0.79	0.69	0.72
ssGBLUP_UPGA10	0.50	0.79	0.46	0.64	0.58	0.37	0.67	0.87	0.60	0.81	0.81	0.83
ssGBLUP_MF4	0.45	0.46	0.40	0.55	0.51	0.36	0.75	0.81	0.63	0.98	0.79	0.93
ssGBLUP_MF10	0.43	0.43	0.39	0.54	0.50	0.32	0.76	0.81	0.66	0.97	0.80	0.87

*SC14, scrotal circumference at 14 months of age; PWG, post weaning gain; WWD, weaning weight direct; WWM, weaning weight maternal; BWD, birth weight direct; BWM, birth weight maternal. ^1^Accuracy of direct and maternal (G)EBV for validation animals was calculated as $\rho_{c,r}=\sqrt{\frac{cov\left({\hat{u}}_{c},{\hat{u}}_{r}\right)}{\left(1-\bar{F}\right)\sigma_{u}^{2}}}$, where cov is the sample covariance; $\hat{\text{u}}$ is the vector of (G)EBV; c and r are subscripts to denote complete and reduced datasets, respectively; $\bar{F}$ is the average inbreeding coefficient for validation animals; and $\sigma_{u}^{2}$ is the direct or maternal additive genetic variance. ^2^Correlation between ${\hat{\text{u}}}_{c}$ and ${\hat{\text{u}}}_{r}$.*

Table 4 also contains stabilities or correlations between (G)EBVs from two successive evaluations (${\hat{\text{u}}}_{r}$ and ${\hat{\text{u}}}_{c}$). Correlations ranged from 0.64 to 0.76 for SC14, 0.02 to 0.87 for PWG, 0.48 to 0.72 for WWD, 0.79 to 0.98 for WWM, 0.66 to 0.81 for BWD, and 0.72 to 0.93 for BWM. Adding genomic information to the model tended to increase the stability of direct and maternal GEBV compared with EBV particularly for SC14, WWD, and BWD. Stabilities were similar for ssGBLUP_UPG4 and ssGBLUP_UPG10 and mostly lower for ssGBLUP_UPGA10 than for ssGBLUP. Further, fitting either four or 10 MFs to ssGBLUP helped increase the stability of GEBV for PWG. Overall, fitting genetic groups had a small impact on the stability of GEBV in the Montana population, except fitting four UPGs to ssGBLUP_UPGA.

### Slope or Dispersion

The slope (b_1_) of the regression of (G)EBVs from the complete dataset on (G)EBVs from the reduced dataset measures the degree of dispersion of (G)EBV estimated under a given model (Table 5). This regression coefficient should be close to one to ensure that there is no inflation or deflation in (G)EBV for validation animals. Regression coefficients ranged from 0.95 to 1.08 for SC14, 0.01 (ssGBLUP_UPG4) to 0.88 for PWG, 0.60 to 0.96 for WWD, 0.51 to 1.15 for WWM, 0.78 to 1.08 for BWD, and 0.79 to 0.99 for BWM. Overall, pedigree-based methods showed no inflation (b_1_ values close to 1.0) for SC14, WWM, and BWD. Conversely, most of the genomic models showed a slight deflation (b_1_values greater than 1) for SC14 and BWD. Overall, PWG showed similar dispersion in models with and without genomic information. Lastly, the inclusion of genomic information considerably reduced the inflation for WWD.

**TABLE 5:** Regression coefficients of direct and maternal (G)EBV from the complete dataset on direct and maternal (G)EBV from the reduced dataset for validation animals and their standard errors (in parentheses) using various models with and without genetic groups.

Model	SC14	PWG	WWD	WWM	BWD	BWM
BLUP	0.95 (0.05)	0.85 (0.04)	0.60 (0.05)	0.92 (0.02)	0.92 (0.05)	0.83 (0.02)
BLUP_UPG4	0.99 (0.06)	0.85 (0.03)	0.62 (0.05)	0.97 (0.01)	0.92 (0.05)	0.83 (0.02)
BLUP_UPG10	0.98 (0.05)	0.85 (0.04)	0.62 (0.05)	0.92 (0.01)	0.93 (0.05)	0.79 (0.02)
BLUP_MF4	0.97 (0.05)	0.84 (0.03)	0.60 (0.05)	0.93 (0.01)	0.93 (0.05)	0.84 (0.01)
BLUP_MF10	0.98 (0.05)	0.84 (0.04)	0.61 (0.05)	0.93 (0.01)	0.94 (0.05)	0.85 (0.01)
ssGBLUP	1.05 (0.05)	0.84 (0.04)	0.96 (0.05)	0.94 (0.02)	1.05 (0.04)	0.95 (0.02)
ssGBLUP_UPG4	1.07 (0.04)	0.84 (0.03)	0.96 (0.05)	0.96(0.01)	1.05 (0.04)	0.97 (0.02)
ssGBLUP_UPG10	1.08 (0.05)	0.84 (0.04)	0.95 (0.05)	0.93 (0.01)	1.08 (0.04)	0.91 (0.02)
ssGBLUP_UPGA4	1.08 (0.04)	0.01 (0.02)	0.93 (0.04)	1.15 (0.04)	0.78 (0.04)	0.99 (0.05)
ssGBLUP_UPGA10	1.03 (0.05)	0.65 (0.02)	0.71 (0.04)	0.51 (0.02)	1.02 (0.03)	0.80 (0.02)
ssGBLUP_MF4	1.04 (0.04)	0.87 (0.03)	0.77 (0.04)	0.99 (0.01)	1.00 (0.04)	0.93 (0.02)
ssGBLUP_MF10	1.06 (0.04)	0.88 (0.03)	0.82 (0.04)	0.96 (0.01)	1.03 (0.04)	0.88 (0.00)

*SC14, scrotal circumference at 14 months of age; PWG, post weaning gain; WWD, weaning weight direct; WWM, weaning weight maternal; BWD, birth weight direct; BWM, birth weight maternal.*

Dispersion differences between pedigree-based models (traditional BLUP, BLUP with UPGs, and BLUP with MFs) were small for all traits (Table 5). Addition of four or 10 UPGs to **A**, **G**, and **A**_22_ in ssGBLUP models (ssGBLUP_UPG4 and ssGBLUP_UPG10) showed only slight changes on inflation (0.01 to 0.04), regardless of the number of added UPGs. When UPGs were added only to A and **A**_22_ in ssGBLUP (ssGBLUP_UPGA4 and ssGBLUP_UPGA10), regression coefficients deviated from 1.0, especially for PWG that had b_1_ equal to 0.01 for ssGBLUP_UPGA4 and 0.65 for ssGBLUP_UPGA10. Those values were both 0.84 when UPGs were fit to **A**, **G**, and **A**_22_. Inflation values for ssGBLUP and ssGBLUP_MF models were very similar (differences of 0.01 to 0.07), except for WWD, which exhibited higher inflation in ssGBLUP_MF models. The MF models (ssGBLUP_MF4 and ssGBLUP_MF10) yielded the smallest dispersions among all ssGBLUP models for PWG, WWM, and BWD. Conversely, the dispersion for BWM was similar across models, except for BLUP_UPG10, which had the greatest inflation.

### Bias

Table 6 shows biases and standard errors of direct and maternal (G)EBV using pedigree-based and genomic models. Biases were calculated as differences between direct and maternal mean (G)EBVs from reduced and complete datasets. These differences have an expected value of zero if (G)EBV are unbiased. The (G)EBV had usually negative biases, indicating that the (G)EBV means for validation animals from the reduced dataset were lower than those from the complete dataset. The (G)EBV biases for maternal traits ranged from −5.34 to 1.92 for WWM and −0.21 to 0.59 for BWM. Most of genomic models with UPGs tended to overestimate maternal (G)EBVs from reduced datasets, resulting in positive biases for WWM.

**TABLE 6:** Biases and standard errors of (G)EBV estimated as differences between direct and maternal mean (G)EBV from reduced and complete datasets using various models with and without genetic groups.

Model	SC14	PWG	WWD	WWM	BWD	BWM
BLUP	−0.13 (0.03)	0.02 (0.09)	−1.29 (0.28)	−0.32 (0.04)	−0.07 (0.05)	−0.05 (0.00)
BLUP_UPG4	−0.15 (0.03)	−0.01 (0.09)	−1.36 (0.28)	−0.17 (0.04)	−0.09 (0.05)	−0.02 (0.00)
BLUP_UPG10	−0.49 (0.03)	−0.19 (0.09)	−1.78 (0.27)	−0.22 (0.04)	−0.09 (0.05)	−0.17 (0.01)
BLUP_MF4	−0.15 (0.03)	0.03 (0.09)	−1.50 (0.28)	−0.21 (0.04)	−0.11 (0.05)	−0.00 (0.00)
BLUP_MF10	−0.17 (0.03)	0.07 (0.08)	−1.45 (0.27)	−0.28 (0.04)	−0.10 (0.04)	−0.03 (0.00)
ssGBLUP	−0.18 (0.03)	−0.08 (0.09)	−1.53 (0.25)	−0.25 (0.04)	−0.07 (0.04)	−0.01 (0.00)
ssGBLUP_UPG4	−0.15 (0.03)	0.01 (0.09)	−1.49 (0.25)	1.92 (0.04)	−0.09 (0.04)	0.59 (0.00)
ssGBLUP_UPG10	−0.48 (0.03)	−0.29 (0.09)	−2.31 (0.25)	0.15 (0.04)	0.01 (0.04)	−0.14 (0.00)
ssGBLUP_UPGA4	0.16 (0.03)	1.16 (0.30)	3.73 (0.25)	0.51 (0.16)	−0.35 (0.05)	−0.18 (0.01)
ssGBLUP_UPGA10	−0.84 (0.04)	−5.66 (0.12)	2.65 (0.28)	−5.34 (0.02)	−0.36 (0.04)	0.00 (0.01)
ssGBLUP_MF4	−0.16 (0.03)	−0.05 (0.08)	1.40 (0.23)	−0.04 (0.03)	−0.25 (0.03)	−0.03 (0.00)
ssGBLUP_MF10	−0.48 (0.03)	−0.09 (0.07)	−2.31 (0.21)	0.36 (0.03)	0.05 (0.04)	−0.21 (0.00)

*SC14, scrotal circumference at 14 months of age; PWG, post weaning gain; WWD, weaning weight direct; WWM, weaning weight maternal; BWD, birth weight direct; BWM, birth weight maternal.*

Overall, genomic models were more biased than pedigree-based models for both direct and maternal traits; however, MF models tended to have similar biases without genomic information. Biases tended to increase when 10 UPGs were added to pedigree-based models. Slight differences in biases for direct traits were observed when four UPGs (ssGBLUP_UPG4) were added to **A**, **G**, or **A**_22_ compared with ssGBLUP. The ssGBLUP_UPGA4 model tended to overestimate (G)EBV for direct traits shown positive bias for SC14, PWG, and WWD. The ssGBLUP_UPGA10 model produced the greatest biases for SC14, PWG, WWM, and BWD but no bias for BWM. The least biased models for WWM and BWM were ssGBLUP_MF4 and BLUP_MF4 or ssGBLUP_MF4, respectively. Overall, biases of genomic models with four MFs were similar to pedigree-based BLUP, lower than ssGBLUP models and genomic models with UPGs.

Increasing the number of UPGs from 4 to 10 in models without genomic information led to larger biases for almost all traits (Table 6). Alternatively, when more UPGs were added to **A**, **G**, and **A**_22_, biases increased for all direct traits, but BWD and the magnitude depended on the trait (e.g., larger biases were observed for SC14 and WWD). Conversely, biases decreased for maternal traits (WWM and BWM). In contrast, models with UPGs in **A** and **A**_22_ showed almost no changes in biases for BWD, although bias decreased for WWD and BWM and increased for SC14, PWG, and WWM. Biases also increased when the number of MFs increased from four to 10, except for BWD.

### Correlations Between (Genomic) Estimated Breeding Values From Different Models and Distribution of (Genomic) Estimated Breeding Value

**FIGURE 1:**
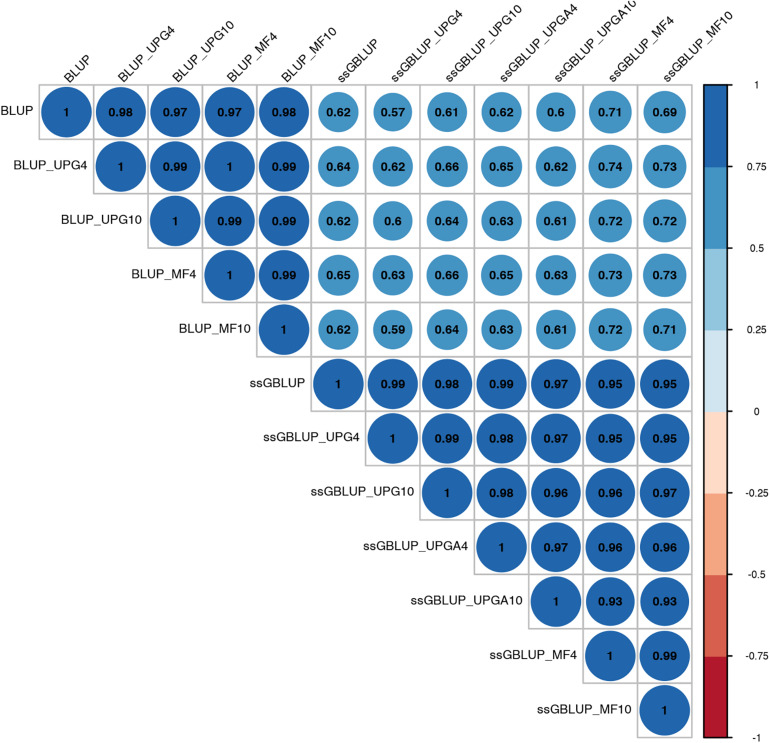
Correlations between breeding values for scrotal circumference at 14 months of age estimated using various models with and without genetic groups.

**FIGURE 2:**
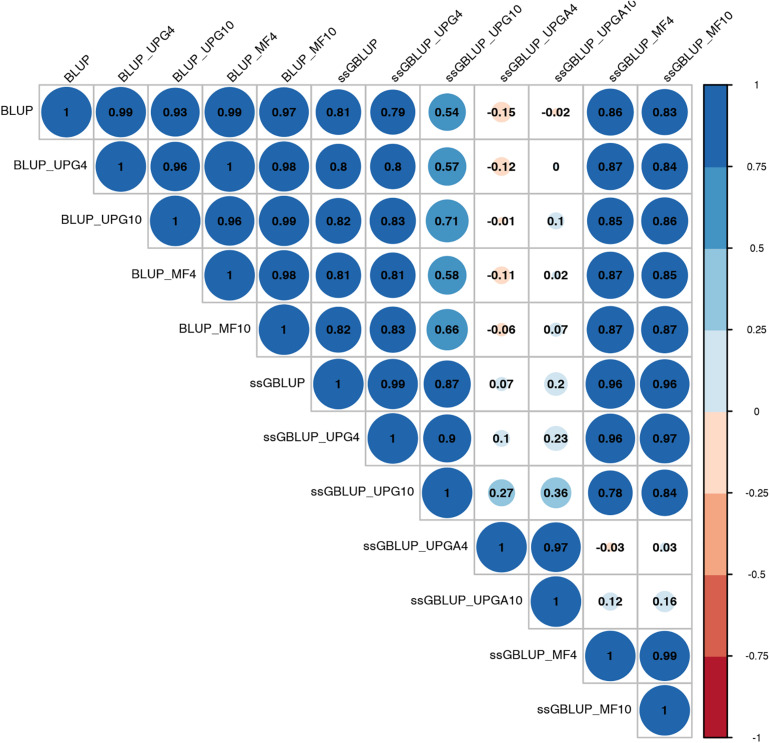
Correlations between breeding values for post weaning weight gain estimated using various models with and without genetic groups.

**FIGURE 3:**
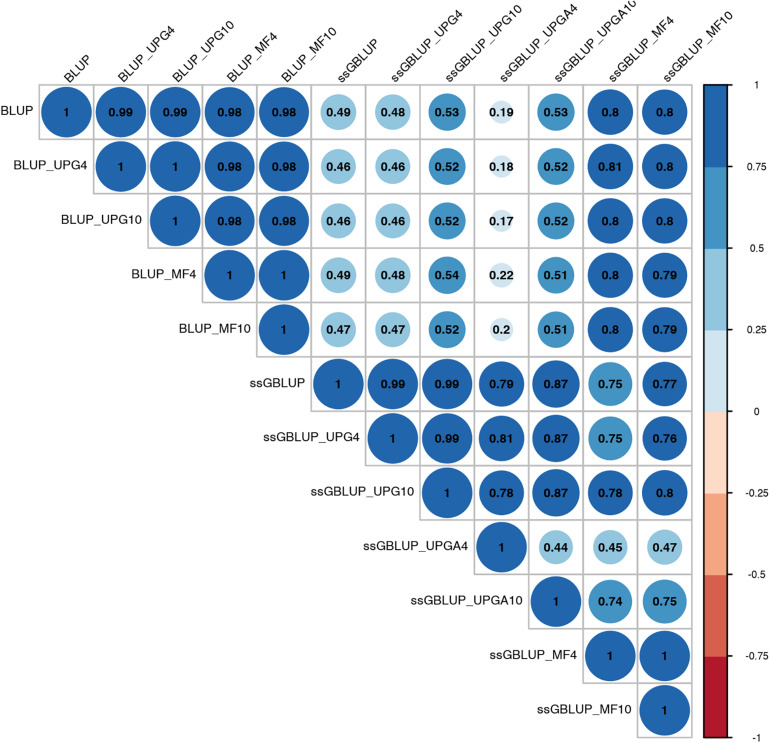
Correlations between breeding values for weaning weight direct estimated using various models with and without genetic groups.

**FIGURE 4:**
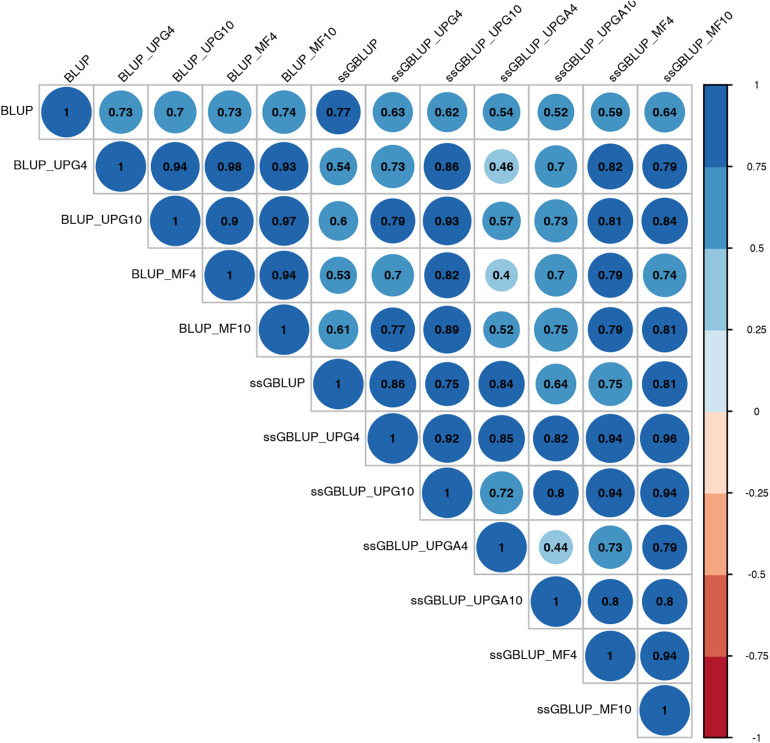
Correlations between breeding values for weaning weight maternal estimated using various models with and without genetic groups.

**FIGURE 5:**
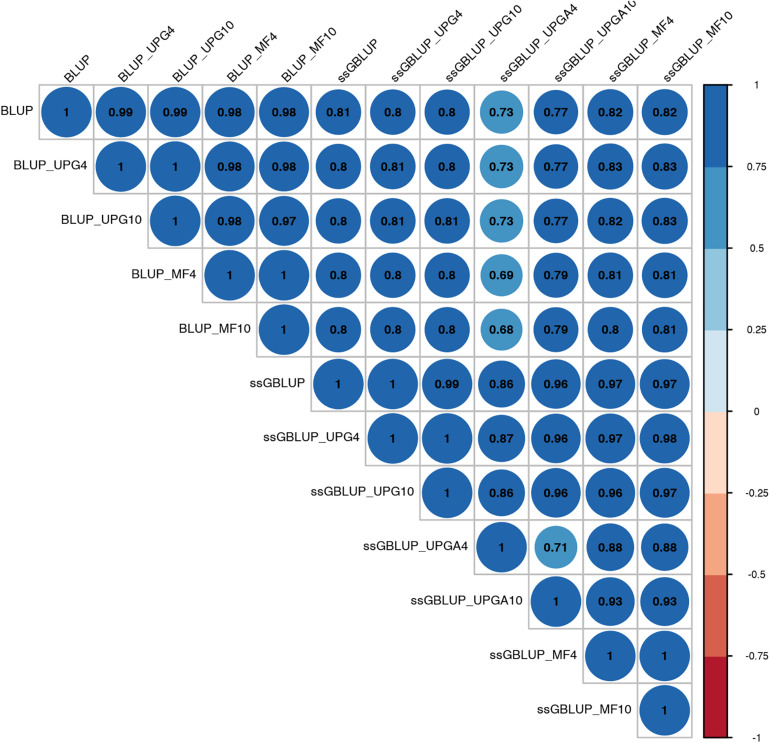
Correlations between breeding values for birth weight direct estimated using various models with and without genetic groups.

**FIGURE 6:**
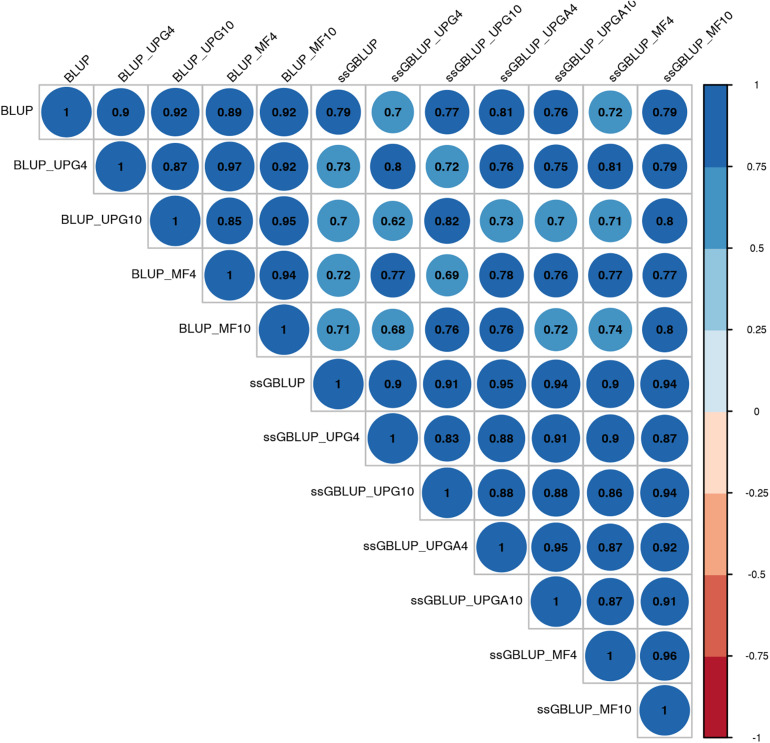
Correlations between breeding values for birth weight maternal estimated using various models with and without genetic groups.

Pearson’s correlation coefficients between (G)EBVs of young animals predicted with various models are presented in Figure 1 for SC14, Figure 2 for PWG, Figure 3 for WWD, Figure 4 for WWM, Figure 5 for BWD, and Figure 6 for BWM. Pearson’s correlation coefficients were used to measure the degree of similarity between (G)EBVs computed using different models. Overall, correlations between (G)EBVs from different models were positive and high, except for (G)EBV from PWG and ssGBLUP_UPGA models. Correlations between (G)EBVs from different models ranged from 0.57 to 1.0 for SC14 (Figure 1). Higher correlations were observed between (G)EBVs within pedigree-based (>0.97) and within genomic models (>0.93). Correlations between pedigree-based EBVs and GEBVs were lower (0.57 to 0.74). Correlations between (G)EBVs from different models ranged from −0.15 to 0.99 for PWG (Figure 2). Correlations between EBVs from pedigree-based models ranged from 0.93 to 1.0, whereas correlations between GEBVs from genomic models fluctuated between −0.03 and 0.99. The lowest correlations were between (G)EBVs from ssGBLUP_UPGA and other models (close to zero). Correlations between (G)EBVs from different models ranged from 0.17 to 1.0 for WWD (Figure 3). The lowest correlations were observed between EBVs from pedigree-based BLUP and GEBV from ssGBLUP_UPGA4. High correlations (∼0.80) between EBVs from pedigree-based and GEBV from genomic models were obtained when MFs were taken into account.

Correlations between (G)EBVs from different models ranged from 0.40 to 0.98 for WWM (Figure 4). The correlations between EBVs from pedigree-based models were higher than 0.70 (0.70 to 0.98), whereas correlations between GEBVs from genomic models ranged from 0.44 to 0.96, and correlations between EBVs from pedigree-based and GEBV from genomic models fluctuated between 0.40 to 0.93. Among genomic models, ssGBLUP_UPGA4 had the lowest correlation with most of models. Correlations between (G)EBVs from different models fluctuated between 0.69 and 1.0 for BWD (Figure 5). The correlations between EBVs from pedigree-based models were close to 1.0 (0.97 to 1.0), correlations between GEBVs from genomic models ranged from 0.71 to 1.0, and correlations between EBVs from pedigree-based and GEBVs from genomic models were around 0.80, except for ssGBLUP_UPA4. Overall, the GEBV from ssGBLUP with MFs had the highest correlations with (G)EBV from other models. Correlations between (G)EBVs from different models ranged from 0.62 to 0.97 for BWM (Figure 6). The correlations between EBVs from pedigree-based models were high (0.85 to 0.97), whereas correlations between GEBVs from genomic models ranged from 0.83 to 0.96, and correlations between pedigree-based and GEBVs from genomic models ranged from 0.62 to 0.82.

**FIGURE 7:**
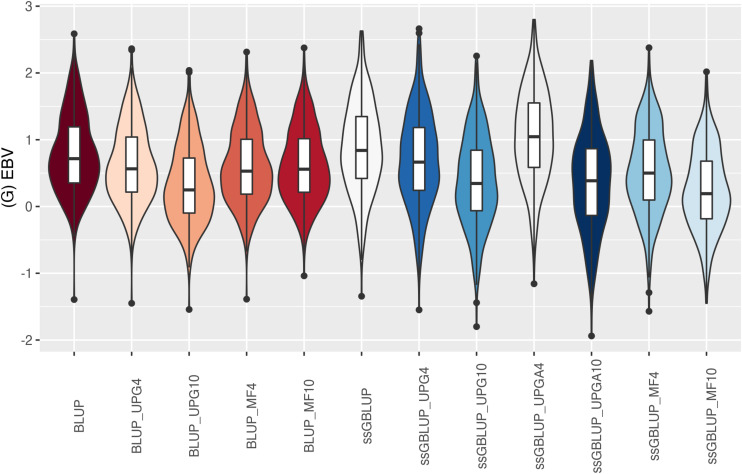
(G)EBVs for validation animals for scrotal circumference at 14 months of age estimated using various models with and without genetic groups. (G)EBVs, (genomic) estimated breeding values.

**FIGURE 8:**
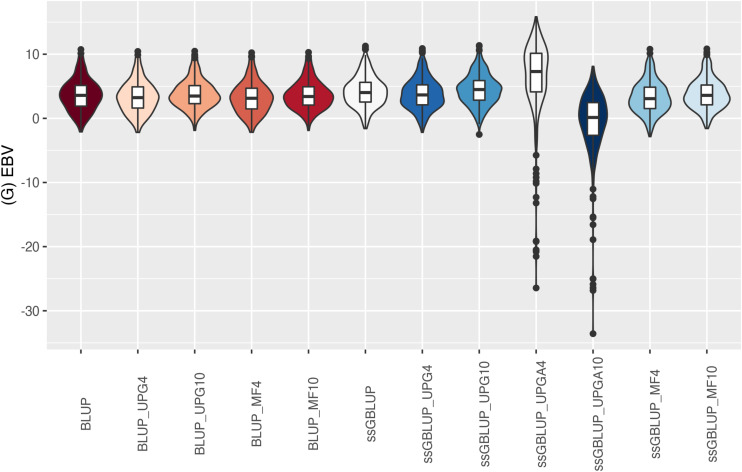
(G)EBVs for validation animals for post weaning weight gain estimated using various models with and without genetic groups. (G)EBVs, (genomic) estimated breeding values.

**FIGURE 9:**
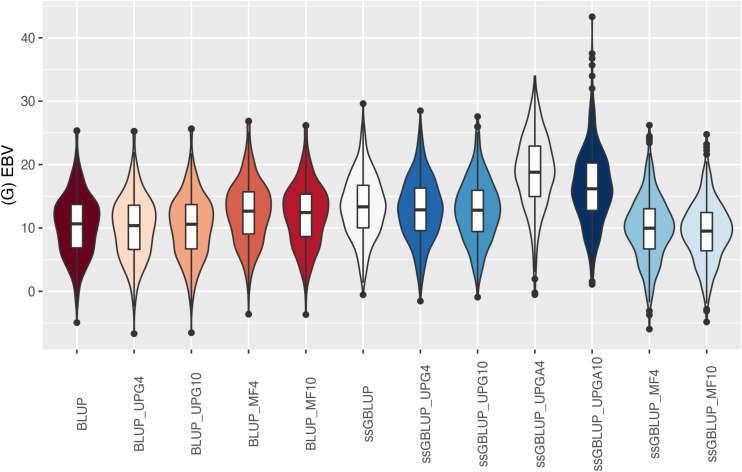
(G)EBVs for validation animals for weaning weight direct estimated using various models with and without genetic groups. (G)EBVs, (genomic) estimated breeding values.

**FIGURE 10:**
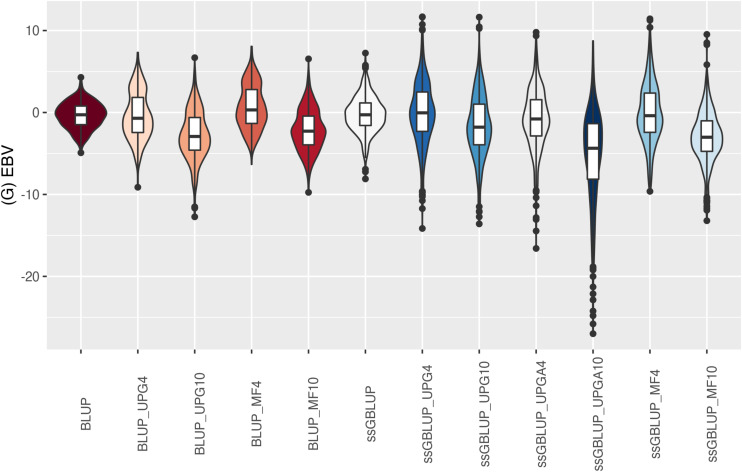
(G)EBVs for validation animals for weaning weight maternal estimated using various models with and without genetic groups. (G)EBVs, (genomic) estimated breeding values.

**FIGURE 11:**
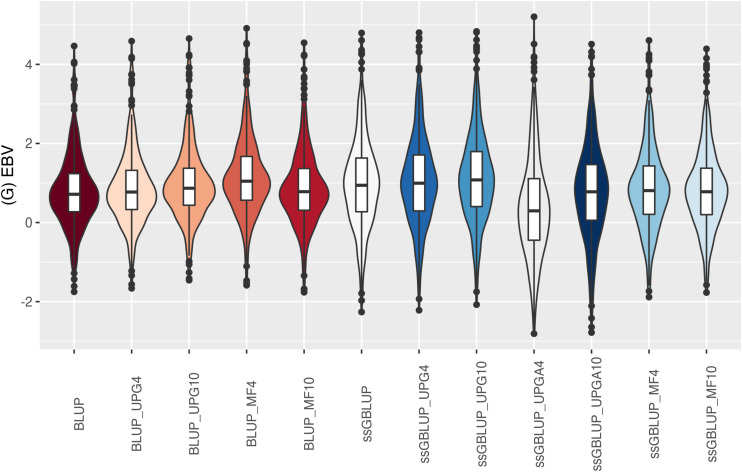
(G)EBVs for validation animals for birth weight direct estimated using various models with and without genetic groups. (G)EBVs, (genomic) estimated breeding values.

**FIGURE 12:**
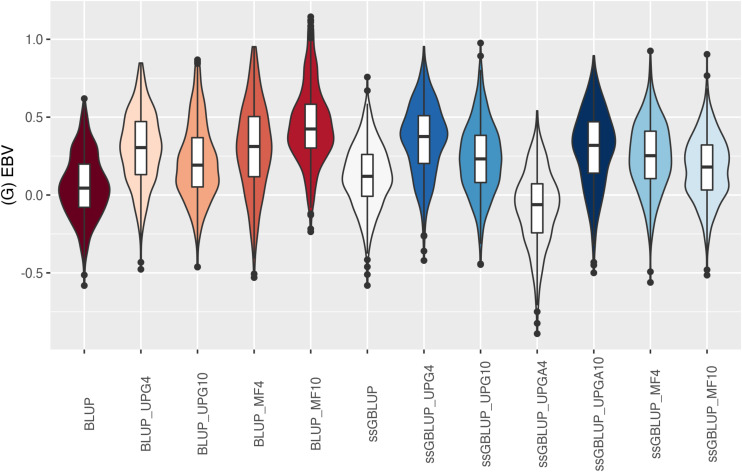
(G)EBVs for validation animals for birth weight direct estimated using various models with and without genetic groups. (G)EBVs, (genomic) estimated breeding values.

The (G)EBV distribution of validation animals predicted with various models is presented in Figure 7 for SC14, Figure 8 for PWG, Figure 9 for WWD, Figure 10 for WWM, Figure 11 for BWD, and Figure 12 for BWM. Overall, violin plots of (G)EBV for SC14 across models were very similar (Figure 7). Conversely, GEBV had more variation than EBV since the frequency of EBV around the median was higher than GEBV around the median. Additionally, more variation of median value was observed among genomic models. The (G)EBV of young animals for PWG (Figure 8) was similar across all models, except for ssGBLUP_UPGA (4 or 10) that produced animals with extreme low GEBV. These models (pedigree-based, ssGBLUP, ssGBLUP_UPG, and ssGBLUP_MF) had most of the (G)EBV values clustered around the median.

The (G)EBV for WWD (Figure 9) had similar distribution among all models, except for ssGBLUP_UPGA10 where animals with extreme high GEBV were reported. Also, the number of UPGs or MFs had almost no impact on the (G)EBV of young animals. Lastly, genomic models with UPGs just in **A** matrices (ssGBLUP_UPGA4 or ssGBLUP_UPGA10) showed more thin distributions and higher median. Among all traits, higher variation of (G)EBV across models was observed for WWM (Figure 10). Overall, EBV had most of the values clustered around the median, whereas GEBV showed more variation within models. Extreme (low or high) (G)EBVs were reported for almost all models, but the frequency of extreme values was higher for ssGBLUP_UPGA models; therefore, these distributions were thinner.

Similar distributions of (G)EBV across models were observed for BWD (Figure 11). Additionally, similar variation across EBV and GEBV themselves, with higher frequency of values around the median for ssGBLUP_MF models, was noticed. Extreme (low or high) (G)EBV values were reported for all models, especially for ssGBLUP_UPGA. Lastly, the median across models was similar. The median of (G)EBV for BWM differed considerably across models (Figure 12). Extreme (G)EBV values were observed for all models. Overall, a higher frequency of (G)EBV around the median was observed for all models, except for ssGBLUP_UPGA4, which had more extreme low values.

## Discussion

Animals in the Montana population must be composed of at least three breeds with a minimum of 12.5% of biological type A and a minimum of 25% of combined biological types N and A. In addition, the maximum percentage of each biological type allowed is 37.5% for group N, 87.5% for group A, and 75% for groups B and C (Santana et al., 2013).

Currently, the genetic evaluation program for the Montana population does not account for missing pedigree. Unfortunately, unless missing pedigree is accounted for, animals with missing parents will have their breeding values regressed toward zero and will likely not be selected as parents of future generations. Unknown genetic groups and MFs can be used to account for missing pedigree and breed structure in base populations. Thus, it is important to assess the ability of UPGs and MFs to account for missing pedigree and breed structure in complex multibreed populations such as the Montana beef cattle population.

### Relationships Within and Across Metafounders (Γ)

Missing pedigree and uncertainty regarding the structure of the base population may be accounted for by adding genetic groups called UPGs based on criteria such as year of birth, generation, breed, line, sex, or a combination of these factors. Recently, Legarra et al. (2015) proposed the use of MFs to account for relationships within and among base populations that are ignored by UPGs. Therefore, the main difference between UPGs and MFs is that the latter accounts for relationships between UPGs, including inbreeding.

Relationships within MFs that are lower than one indicate negative inbreeding, which implies a higher frequency of heterozygotes relative to the average of the population. This also means that the base population has a large amount of genetic variability. Conversely, when these values are higher than one, they indicate that base populations are inbred (Legarra et al., 2015). Additionally, positive relationships between MFs indicate that ancestor populations overlap, negative values indicate population divergence, and zero values indicate that base populations are unrelated. Estimates of **Γ** different from zero permit full consideration of MFs in genetic evaluation, whereas a zero **Γ** is equivalent to having UPGs for **A** and **A**_22_ (Bradford et al., 2019).

Relationships within MFs were lower than one, whereas relationships between MFs were different from zero in the Montana population. Similar findings were reported in simulated and real datasets (Xiang et al., 2017; van Grevenhof et al., 2018; Bradford et al., 2019). We found negative relationships between purebred MFs (N and B, and N and C) when using 10 MF groups. This could be explained by the fact Montana is composed of two subspecies of *Bos taurus*, i.e., *B. taurus indicus* in group N and *B. taurus taurus* in groups A, B, and C. However, negative relationships were not observed when using only four MF groups, possibly because intermediate crosses were implicitly nested within the four biological types. This created stronger ties among the biological types based on SNP information, hence the positive relationship coefficients between MF groups.

The relationship coefficients within MFs in our study were similar to those reported by other authors. In a simulation study, van Grevenhof et al. (2018) reported relationships within MFs of 0.17 for related parental lines and of 0.74 for unrelated parental lines. Bradford et al. (2019) obtained relationships within MFs ranging from 0.54 to 0.71 in a simulated dairy cattle population. Colleau et al. (2017) found relationships within MFs of 0.47 in sheep, and Legarra et al. (2015) computed relationships of 0.55 for Holsteins and 0.77 for Jerseys. In general, the relationships within groups in MF10 were higher than in MF4, implying more variability in the latter. This could be explained by the method used to form groups in each scenario. Animals in MF4 with a higher proportion of a certain biological type (N, A, B, and C) were allocated to a purebred group in such a way that these purebred groups were not homogeneous (i.e., MF4 groups were composed of purebred and crossbred animals).Conversely, the strategy for MF10 was to assign purebred and crossbred animals to various groups that were more homogeneous.

### Accuracy and Stability of (Genomic) Estimated Breeding Value

Adding genomic information increased the accuracy of GEBV for all traits, except for PWG. Genomic information increases the accuracy of estimation of relationship coefficients between animals as well as the accuracy of estimation of Mendelian sampling terms, which is not possible through the pedigree-based relationship matrix (**A**). For instance, two unrelated individuals in **A** will be related through **H** if these animals are related through the **G** matrix, even if the pedigree does not show it (Legarra et al., 2014). When animals have neither phenotypes nor progeny, their GEBV is composed of the sum of one half of the (G)EBV of their parents, genomic information coming from **G**, and pedigree information coming from **A**_22_ (Lourenco et al., 2015a). On the other hand, the EBV of ungenotyped young animals without phenotypes and progeny is composed of the sum of one half of the (G)EBV of their parents plus the Mendelian sampling (Quaas, 1988). Thus, taking genomic information into account permits the inclusion of more information to estimate breeding values for young animals without records and progeny. How much this additional information contributes to the improvement of EBVs can be measured through their increase in accuracy by using models with marker information.

Increases in accuracy of models’ prediction by using genomic information were reported in numerous studies, supporting the benefits of genomic selection in livestock breeding programs (Aguilar et al., 2010; Chen et al., 2011; Baloche et al., 2014; Garcia et al., 2018). However, increases in accuracy of prediction depend on a variety of factors that can differ among traits. Genomic information produced either low or no increase in accuracy for PWG, perhaps due to the small size of the Montana reference population and the number of sires with progenies in the reference population. These factors need to be taken into account when establishing genomic selection schemes in livestock populations. A good way to choose genotyping strategies aiming to increase GEBV accuracies would be to include older animals with high EBV accuracies and large numbers of progeny (Lourenco et al., 2015b). This was a limiting factor in the Montana population because its reference population contained only a few animals with low EBV accuracies, particularly for PWG.

The inclusion of either four or 10 UPGs in all ssGBLUP relationship matrices was unable to markedly increase GEBV accuracies; thus, only slight differences among these genetic evaluation models and ssGBLUP were observed. The ssGBLUP_UPGA4 model yielded higher GEBV accuracies than other genomic models for WWD, and ssGBLUP_UPGA10 yielded higher GEBV accuracies than other genomic models for PWG and WWM. In addition, increasing the number of UPGs may not always be the best approach because UPG estimates are related to numbers of animals and phenotypes in each UPG group (Tsuruta et al., 2014).

The GEBV accuracies were more stable when UPGs were added to **A**, **G**, and **A**_22_matrices (ssGBLUP_UPG). The number of UPGs appeared to have a higher impact on GEBV accuracies when they were not added to the **G** matrix. There is some evidence that it is not necessary to add UPGs to **G** in purebred populations because this matrix is not affected by missing pedigree. However, matrix **G** can be affected by line or breed differences (Plieschke et al., 2015); thus, UPGs should be included in the **G** matrix used in composite populations such as the Montana beef cattle population.

The main reason to account for UPGs in genetic evaluation is that genetic trends could have large biases when genetic differences among groups are ignored, particularly in strongly selected populations. Conversely, poor definition and incorrect assignment of UPGs can also introduce biases. Therefore, UPGs need to be estimated accurately with sufficient information to avoid these issues (Tsuruta et al., 2019). However, when the goal is to predict the EBVs of animals from the youngest generation, removing UPGs from the model should not have a large impact on EBV accuracies when genotyped animals have no missing parents (Misztal et al., 2013) as reported in our study [minor differences in (G)EBV accuracy between models with and without UPGs].

Our results indicated that MFs may not improve GEBV accuracies. Similar results were reported in previous studies with simulated and real datasets. In a simulation study, Bradford et al. (2019) showed that GEBV accuracies were more related to trait heritabilities than inclusion of MFs or UPGs in ssGBLUP. These authors indicated that traits with higher heritabilities had higher (G)EBV accuracies than traits with lower heritabilities even with pedigree-based models. This occurs because accuracy of (G)EBV is a function of heritability. These authors also found a slight increase in GEBV accuracies in ssGBLUP with MFs (0.01 to 0.04 for traits with a heritability of 0.3 and 0.01 to 0.03 for traits with a heritability of 0.1). In contrast, we found lower GEBV accuracies for ssGBLUP models with MFs, although GEBVs from these models were still accurate.

Two separate simulation studies found either a small increase in GEBV accuracy (0.02; Garcia-Baccino et al., 2017) or no difference in GEBV accuracy between ssGBLUP models with and without MFs (van Grevenhof et al., 2018). Xiang et al. (2017), using data from purebred and crossbred Landrace and Yorkshire pigs, showed that the inclusion of MFs in ssGBLUP performed as well as ssGBLUP with breed of origin of alleles, which requires phasing genotypes and can be done in a simple way. MFs were developed to make the **G** and **A** matrices compatible. MFs are only applied to the **A** matrix; thus, incompatibilities between the **G** and **A** matrices are related to differences in base populations. However, this issue is more related to bias than to accuracy or stability of GEBVs.

The stability of GEBV was measured as the correlation between GEBVs from two consecutive evaluations, one with the complete dataset and another one with a reduced dataset. Stability can be interpreted as the ability of the reduced dataset to predict the complete dataset. Overall, the inclusion of genomic information in ssGBLUP models helped increase the correlations between GEBVs from the reduced and complete data, except for PWG. No changes in stability were expected by adding MFs to ssGBLUP models. However, slight differences in stability between GEBVs from ssGBLUP_MF and ssGBLUP were observed for SC14, WWD, and BWD. Conversely, the inclusion of MFs helped to get higher correlations for PWG in ssGBLUP models than in pedigree-based models. These results indicate that MFs could help increase GEBV accuracies for traits when ssGBLUP models without MFs yield accuracies and correlations similar to those of pedigree-based models.

### Slope or Dispersion

When the slope of the regression of (G)EBV from the complete dataset on (G)EBV from the reduced dataset is equal to 1, both sets of (G)EBVs are on a similar scale. Inflation occurs when b_1_ is lower than 1, and deflation happens when b_1_ is larger than 1. The scale of (G)EBV is a key component in selection schemes because it permits a fair comparison of (G)EBV among animals, and consequently proper selection decisions (Piccoli et al., 2018). Inflation causes over dispersion, which is detrimental to genomic predictions, especially when selection candidates are from different generations or have different amounts of information (Neves et al., 2012).

Inclusion of genomic information helped reduce the inflation of GEBV for WWD. However, inclusion of UPGs in all relationship matrices (ssGBLUP_UPG) or MFs (except for WWD) had only a small impact on GEBV dispersion across traits. Bradford et al. (2019) showed that the dispersion of EBV from BLUP models that did not account for missing pedigree could be greater than that of genomic models. Inflation ingenomic models is likely caused by a mismatch between the scale of the pedigree and genomic relationship matrices (Misztal et al., 2017). Vitezica et al. (2011) showed that inflation is related to the heritability of the trait and selection pressure. According to Tsuruta et al. (2019), the inflation of GEBV can be reduced by weighting ${\text{A}}_{22}^{-1}$ by a factor smaller than 1.0 or by reducing the additive genetic variance of the trait. Dispersion of GEBV from ssGBLUP may also be observed when the pedigree is deep but incomplete, and when inbreeding is not considered in **A**. Consequently, inflation/deflation can be reduced by a combination of pedigree truncation, incorporation of inbreeding in **A**, and accounting for inbreeding of unknown parents (Misztal et al., 2017).

Our results showed a small or no change in inflation/deflation of (G)EBV when UPGs were added to BLUP and ssGBLUP models, except when those were removed from **G** matrix (ssGBLUP_UPGA), especially for PWG regression coefficients that deviated from 1.0, especially for PWG that had b_1_ equal to 0.01 for ssGBLUP_UPGA4. This occurs because UPG solutions for PWG and ssGBLUP_UPGA4 from reduced and complete data are very different (Table 2). Conversely, the inclusion of genomic information eliminated the inflation for WWD. Animals with and without phenotypes sharing the same UPGs must be related to appropriately estimate UPG effects. Similarly, if animals in **A**^22^ (block of **A**^−1^for genotyped animals) and **A**^11^ (block of **A**^−1^ for non-genotyped animals) are unrelated (i.e., **A**^12^ = 0), **H**^−1^ will not contribute to the estimation of UPG effects (Tsuruta et al., 2019). The inclusion of MFs in genomic models helped to alleviate the inflation of (G)EBV for PWG, WWM, and BWD. The estimation of MFs effects relies on **Γ** and, consequently, on the number of genotyped animals with phenotypes connected to each MF.

The inflation/deflation of GEBVs from ssGBLUP_UPG was lower than that of GEBVs from ssGBLUP_UPGA. This raises the question of whether UPGs should be added to **G**, given that genomic relationships do not rely on pedigree missingness. Matrix **G** accounts for line and breed differences (Plieschke et al., 2015); thus, adding UPGs to this matrix may be beneficial in crossbred and multibreed populations. Results from this study indicate that addition of UPGs to **G** is important to obtain (G)EBVs with the smallest inflation/deflation in the Montana composite beef cattle population. However, this study was based on a small number of genotyped animals; thus, additional research witha larger Montana dataset would be needed to confirm our findings.

### Bias

Bias is defined as the ability to correctly predict the mean breeding value of selection candidates (Granado-Tajada et al., 2020). Nonzero biases compromise our ability to correctly estimate genetic trends and genetic gains (Legarra and Reverter, 2018). Negative biases indicate underestimation of (G)EBV from the reduced dataset. Negative biases existed for all models and traits in our study, with overall stronger biases for ssGBLUP models with and without UPGs than for BLUP models. Bias estimates were similar for BLUP and ssGBLUP_MF models for all traits, except for WWD. For SC14, PWG, and BWM, the ssGBLUP_MF4 was similar to BLUP. Conversely, for BWD, the BLUP and ssGBLUP_MF10 models yielded similar bias, while for WWM, the ssGBLUP_MF4 model yielded the lowest biased GEBV. This indicates that the inclusion of four MFs in ssGBLUP models would likely reduce biases in genomic models to the same extent or even more than BLUP models, although the latter could still yield biased EBV due to model artifacts and preselection of animals. A way to eliminate GEBV biases in ssGBLUP models in composite populations would likely involve a minimally biased pedigree-based model, and addition of genomic information together with MFs to model heterogeneity in the base population. Because artificial selection can generate biases due to an increase in the genetic level and a reduction in the additive genetic variance, finding an unbiased model could be challenging (Legarra and Reverter, 2018).

Bradford et al. (2019) found no (G)EBV biases in pedigree-based and genomic models with complete pedigree in a simulation study. These authors also reported an increase in EBV biases from pedigree-based models when UPGs were used to account for missing pedigree, in agreement with our findings in the Montana population. Biases resulting from the inclusion of UPGs are primarily due to inaccurate estimates of UPG effects, which reinforces the importance of a robust group definition. Tsuruta et al. (2014) showed that combining groups with small amounts of information helped to reduce GEBV biases in the US Holstein population.

Bradford et al. (2019) found larger biases in pedigree-based models than in genomic models when missing pedigree was unaccounted for in a simulation study, in contrast to results from the Montana population. However, our results are in agreement with those of Garcia-Baccino et al. (2017), who found larger GEBV biases in ssGBLUP than in BLUP models; the latter was in fact unbiased. However, these authors used simpler models than the ones with direct and maternal additive genetic effects, maternal permanent environmental effects, and multiple fixed effects used in this study. In addition, GEBV biases in this study were likely influenced by the complex structure of the Montana composite beef cattle population.

### Correlations Between (Genomic) Estimated Breeding Values From Different Models and Distribution of (Genomic) Estimated Breeding Value

Correlations between (G)EBVs from different models represent the degree of similarity between (G)EBVs across models. The high degree of similarity between EBVs from the various pedigree-based models for all traits indicates that the inclusion of UPGs and MFs produced minor changes in EBV values. Overall, correlations between GEBVs from genomic models were lower than correlations between EBVs from BLUP models, indicating larger changes in GEBV than in EBV. Correlations between (G)EBVs from BLUP and ssGBLUP_MF models were greater than between (G)EBVs from BLUP and ssGBLUP models with or without UPGs. The (G)EBV from UPGs and MF models included group effects; thus, changes in (G)EBV from these models depended on the accuracy of group effect estimates. The low correlations between (G)EBVs from ssGBLUP_UPGA and (G)EBV from other models, particularly for PWG and WWD, indicate inaccurate estimates of UPG effects for these traits. Additionally, when animals with and without phenotypes in the same group are unrelated, group effects are not estimable; thus, the ssGBLUP_UPGA model becomes equivalent to the ssGBLUP model without UPGs. Furthermore, if genotyped animals are not related to non-genotyped animals (animals in **A**^22^ and **A**^11^, respectively), **H**^−1^ will not contribute to the estimation of group solutions, which is also equivalent to ignoring UPGs (Tsuruta et al., 2019). Lastly, because estimates of UPG effects are trait-dependent, changes in (G)EBV from pedigree-based and genomic UPG models also depend on the evaluated traits.

## Conclusion

Genomic information helped improved the accuracy and persistence of predictions in the Montana composite beef cattle population. Addition of UPGs either to only the pedigree relationship matrix or to both the pedigree and genomic relationship matrices in ssGBLUP models to account for missing pedigree and base population heterogeneity did not improve accuracy, inflation/deflation, and bias of genomic predictions. Thus, addition of UPGs to relationship matrices in ssGBLUP models is not recommended in this population. Although the addition of MFs to ssGBLUP models was unable to increase the accuracy, this model yielded GEBV with lower inflation/deflation for some traits and the least biased genomic predictions. Therefore, this model could be recommended for genomic evaluation in small composite beef cattle populations.

## Data Availability Statement

The data cannot be made publicly available, because it is property of the Montana Tropical Composite breeders and this information is commercially sensitive. Reasonable requests for access to the datasets for research purposes can be e-mailed to JF, jbferraz@usp.br.

## Ethics Statement

Animal Care and Use Committee approval was not obtained for this study because data set was provided by existing database.

## Author Contributions

SK, YM, FB, and DL conceived and designed the project. JE and JF organized sample collection and genotyping. SK, YM, and DL analyzed the data. SK, YM, ST, and DL discussed the results. SK wrote the manuscript. DL and ST edited the manuscript. All authors contributed to manuscript revision and read and approved the submitted version.

## Conflict of Interest

The authors declare that the research was conducted in the absence of any commercial or financial relationships that could be construed as a potential conflict of interest. The reviewer AR declared a past co-authorship with one of the authors to the handling editor.

## Publisher’s Note

All claims expressed in this article are solely those of the authors and do not necessarily represent those of their affiliated organizations, or those of the publisher, the editors and the reviewers. Any product that may be evaluated in this article, or claim that may be made by its manufacturer, is not guaranteed or endorsed by the publisher.
